# Syndecan-1 (CD138) deficiency increases *Staphylococcus aureus* infection but has no effect on pathology in a mouse model of peritoneal dialysis

**DOI:** 10.1186/s12929-016-0232-9

**Published:** 2016-02-01

**Authors:** Paulina M. Kowalewska, Uyen T. Nguyen, Lori L. Burrows, Alison E. Fox-Robichaud

**Affiliations:** Thrombosis and Atherosclerosis Research Institute and the Department of Medicine, McMaster University, Hamilton, ON Canada; Department of Biochemistry and Biomedical Sciences and the Michael G. DeGroote Institute for Infectious Diseases Research, McMaster University, Hamilton, ON Canada; David Braley Cardiac, Vascular and Stroke Research Institute, C5-106, 237 Barton Street East, Hamilton, ON L8L 2X2 Canada

**Keywords:** Intravital microscopy, Leukocyte recruitment, Microcirculation, Fibrosis, Peritonitis, Proteoglycans

## Abstract

**Background:**

Technique failure in peritoneal dialysis (PD) due to fibrosis and angiogenesis is complicated by peritonitis. *Staphylococcus aureus* infection is one of the most common causes of peritonitis in PD. The heparan sulfate proteoglycan, syndecan-1 (CD138), was reported to regulate fibrosis, angiogenesis, inflammation and *S. aureus* infection. The objectives of this study were to examine the effects of syndecan-1 on *S. aureus* infection and histopathology in a PD model.

**Results:**

Syndecan-1^-/-^ and wild type mice were dialyzed for 4 weeks and infected intraperitoneally with *S. aureus*. Tissues were collected after 4 h for histomorphometric analysis. Intravital microscopy was used to observe leukocyte recruitment and to quantify syndecan-1 in the parietal peritoneum microcirculation. The dialyzed syndecan-1^-/-^ mice were more susceptible to *S. aureus* infection than undialyzed syndecan-1^-/-^ controls and wild type animals. However, peritoneal fibrosis and neovascularization due to PD did not differ between syndecan-1^-/-^ and wild type mice. Intravital microscopy showed that in *S. aureus* infection, syndecan-1 was removed from the subendothelial layer of peritoneal venules but syndecan-1 deficiency did not affect leukocyte recruitment.

**Conclusions:**

This study indicates that, while syndecan-1 is important for providing a barrier to acute *S. aureus* infection in PD, it does not affect peritoneal fibrosis and angiogenesis.

## Background

Peritoneal dialysis (PD) is an effective replacement therapy for chronic kidney failure. In PD, dialysis solution is administered into the peritoneal cavity through a surgically implanted catheter and there is an exchange of water and solutes between the patient’s blood and the indwelling solution across the peritoneal membrane, allowing for removal of excess water and wastes. However, PD failure typically occurs because of infection or loss of ultrafiltration [1–3]. Underlying the loss of ultrafiltration are deleterious alterations of the peritoneum involving progressive fibrosis, angiogenesis, vasculopathy, and phenotypic change of mesothelial cells of the peritoneal membrane [4–6] to a fibroblast-like state, a process referred to as epithelial-mesenchymal transition (EMT). These deleterious alterations are further complicated by bacterial peritonitis. The main site of infection in PD is the lumen of the catheter from touch-contamination as well as the outside of the catheter at the exit site. *Staphylococcus aureus*, *Staphylococcus epidermidis*, *Pseudomonas aeruginosa* and *Escherichia coli* are among the most common microorganisms causing peritonitis in PD [7–9].

The cell surface heparan sulfate proteoglycan, syndecan-1 (Sdc1; CD138), modulates diverse processes such as inflammation [10], wound repair [11], angiogenesis [12], fibrosis [13], and EMT [14, 15]. Syndecan-1 is a single-pass type I transmembrane protein with several heparan sulfate glycosaminoglycan chains covalently attached to the distal portion of the extracellular domain. Syndecan-1 is expressed on mesothelial cells of the parietal peritoneum, and in the subendothelial compartment of peritoneal venules [16]. Some microbial pathogens subvert syndecan-1 to increase dissemination and host defense evasion during infection. Among the best-characterized pathogens that manipulate syndecan-1 is *S. aureus* [17]. Although the role of certain proteoglycans was examined in dialysis [18], the role of syndecan-1 has not been investigated in PD. Given that syndecan-1 regulates inflammation, fibrosis, angiogenesis and EMT, and syndecan-1-microbial interactions are important in the pathogenesis of *S. aureus*, the aims of this study were to characterize the effects of syndecan-1 deficiency on peritoneal histopathology, foreign body reaction, and *S. aureus* infection during subacute (4 weeks) PD. In addition, we examined the effects of acute *S. aureus* infection on syndecan-1 protein expression in the peritoneal microcirculation and its role in leukocyte recruitment.

## Methods

### Animals

The animal protocols met the regulations set by the Canadian Council of Animal Care and were approved by the McMaster University Animal Research Ethics Board (Animal Utilization Protocol #11-01-03). All animal protocols followed the “Animal research: reporting of *in vivo* experiments” guidelines [19]. Six- to 8-week-old male BALB/c mice were obtained from Taconic (Germantown, NY, USA) and given at least 1 week to acclimatize in a specific pathogen-free facility. These mice were verified to be *S. aureus*-free by the supplier. The age-matched syndecan-1 null (*Sdc1*^-/-^) male mice used in this study were on a BALB/c background and sourced from a colony that was interbred and maintained at McMaster University Central Animal Facility. The *Sdc1*^-/-^ breeders, which tested negative for *S. aureus*, were a kind gift from Dr. Pyong W. Park (Children’s Hospital, Harvard Medical School, Boston, MA, USA). Syndecan-1 deficiency in the mouse colony was confirmed using *in vivo* imaging of the parietal peritoneum microcirculation with immunofluorescence [16].

### Nonuremic subacute peritoneal dialysis model

Detailed methods were previously described [20]. Sterile silicone catheters (inner diameter (ID) 0.635 mm, outer diameter (OD) 1.1938 mm) attached to silicone injection ports (Penny MousePort™; Access Technologies, Skokie, IL, USA) were aseptically implanted subcutaneously into wild type and *Sdc1*^-/-^ mice under gaseous anesthesia. Before insertion, the port and catheter were flushed with 10 % heparin (1000 USP Units mL^-1^; Sandoz Canada Inc., Boucherville, QC, Canada) to maintain patency. The 1-cm tip of the catheter was inserted through the right lower quadrant of the abdominal wall into the peritoneal cavity. One week after catheter implantation, 10 % heparin solution was injected into the port to keep the catheter patent. The mice were given 2 weeks to acclimatize to the implant. After the acclimatization period, daily 2-mL injections of a conventional lactate-buffered dialysis solution (Dianeal PD4 CAPD Solution with 2.5 % [w/v] dextrose and 2.5 mEq L^-1^ calcium, approximate pH 5.2; Baxter, Mississauga, ON, Canada) were administered into the Penny MousePort™ for 4 weeks. The skin over the port was sterilized and injections were performed using aseptic technique in a biological safety cabinet.

### Bacterial strain and growth conditions

*S. aureus* H1559 cultures were grown overnight on a shaker in tryptic soy broth (TSB) (EMD, Darmstadt, Germany) plus 15 μg mL^-1^ of erythromycin (Sigma-Aldrich®, St. Louis, MO, USA) to select for a green fluorescent protein (GFP)-expressing plasmid, constitutively expressed from the *prsA* promoter cloned into pCN56 [21]. The overnight cultures were diluted 100-fold in fresh TSB and grown for 2.5 h at 37 °C to an optical density of ~0.5 at 600 nm. The subcultures were sedimented, washed and suspended in 100 μL of sterile 1X phosphate-buffered saline (PBS; pH 7.2, 0.14 M NaCl, 2.7 mM KCl, 0.88 mM KH_2_PO_4_, 6.4 mM Na_2_HPO_4_ · 7H_2_O). Colony-forming units (CFU) were enumerated following serial dilutions and plating on tryptic soy agar (TSA) plates with 10 μg mL^-1^ erythromycin (Teknova, Hollister, CA, USA). The inocula contained approximately 1.8 × 10^8^ CFU.

### *S. aureus* infection and tissue collection

On the last day of the 4-week dialysis period, mice received an injection of *S. aureus* into the subcutaneous port, which was immediately followed by an injection of 2 mL of dialysis solution through the port. The infected non-PD controls were wild type and *Sdc1*^-/-^ animals that did not undergo dialysis and were injected intraperitoneally (IP) on the right side with an equivalent amount of *S. aureus* suspension. After 4 h, wild type and *Sdc1*^-/-^ mice were anesthetized with a subcutaneous injection of a mixture of ketamine (200 mg kg^-1^) and xylazine (10 mg kg^-1^). Under sterile conditions, peritoneal lavage was performed with 2 mL of PBS. The intra-abdominal portion of the PD catheter was retrieved and fixed in 2 % [v/v] glutaraldehyde in sodium cacodylate. Samples of the anterior abdominal wall from dialyzed animals were collected in a standardized manner from the left upper quadrant, adjacent to the midline and contralateral to the catheter insertion site. This position was above the region that contacted the intra-abdominal catheter tip. For the undialyzed animals that received a suspension of *S. aureus* with an IP needle injection, the section of abdominal wall sampled was contralateral and superior to the injection site. Anatomically matching areas were extracted for baseline histology. The samples were fixed in 10 % [v/v] buffered formalin. Additional samples of the anterior abdominal wall, taken from the left lower quadrant, were homogenized. Blood was collected via cardiac puncture. Euthanasia was ensured by cervical dislocation.

Colony-forming units from the peritoneal lavage fluid, blood and abdominal wall homogenate were enumerated after serial dilutions and plating on TSA plates with 10 μg mL^-1^ erythromycin. The peritoneal lavage fluid and blood were mixed with 3 % [v/v] acetic acid (Caledon Laboratory Chemicals, Georgetown, ON, Canada) and 1 % [w/v] crystal violet (Sigma-Aldrich®) in a 5:44:1 ratio. Blood cell counts with a hemacytometer were averaged from 6 separate samples of the mixture of blood or lavage fluid. Differential white blood cell counts were performed on smears of blood fixed in methanol and stained with eosin and thiazine (Harleco Hemacolor® stain set; EMD Chemicals, Gibbstown, NJ, USA). Results were compared with baseline levels in wild type and *Sdc1*^-/-^ mice that did not undergo PD and were not infected. In total, 6 groups were observed, with *n* = 8 mice in the wild type and *Sdc1*^-/-^ baseline groups as well as the *Sdc1*^-/-^ infected non-PD group and *Sdc1*^-/-^ infected PD group. The wild type infected non-PD and infected PD groups both had 14 mice.

### Scanning electron microscopy

The catheter pieces were immersed for 2 h in primary fixative solution (2 % [v/v] glutaraldehyde in 0.1 M sodium cacodylate buffer at pH 7.4). The samples were rinsed twice in buffer solution and post-fixed for 1 h in 1 % [w/v] osmium tetroxide in 0.1 M sodium cacodylate buffer. After the second fixation step, the samples were dehydrated through a graded ethanol series and then dried in a critical point dryer. After drying, the catheter pieces were cut open to expose the inner surface of the catheters and mounted onto scanning electron microscopy stubs. The stubs were sputter coated with gold and viewed with a Tescan Vega II LSU scanning electron microscope (Tescan USA, Cranberry TWP, PA, USA) operating at 20 kV. Image acquisition was performed with Tescan VegaTC operating software (Tescan USA, Cranberry TWP, PA, USA).

### Histopathologic examination

The formalin-fixed tissue samples were embedded in paraffin and thin-sectioned. The cross-sections were stained with hematoxylin and eosin (H&E) (Sigma-Aldrich®) or picro-sirius red (abcam®, Cambridge, MA, USA) for visualization of collagen. For identification of elastic fibers, cross-sections were stained with resorcin-fuchsin and counterstained in van Gieson’s solution and Weigert’s iron hematoxylin (Electron Microscopy Sciences, Hatfield, PA, USA). Microscopic examination was performed using an Olympus BX41 microscope with an Olympus DP72® camera (Olympus America Inc., Center Valley, PA, USA) and acquired using SlideBook 5.0 custom-built microscopy software (Intelligent Imaging Innovations, Inc., Denver, Co, USA). Peritoneal thickness was measured at five random locations in each H&E-stained section and averaged, and the number of peritoneum-associated blood vessels was counted along the entire length of the section of peritoneum and recorded as vessels per mm of peritoneum. The blood vessel counts were verified using immunohistochemical staining of the paraffin-embedded tissues with a monoclonal antibody to the cell surface marker platelet endothelial cell adhesion molecule-1 (PECAM-1; CD31).

### Immunohistochemistry

Paraffin sections were placed on a slide and deparaffinised, hydrated and heated in citrate buffer in preparation for immunohistochemistry (IHC). After quenching endogenous peroxidase activity and blocking with normal goat serum, the sections were incubated with rat anti-mouse F4/80 monoclonal antibody (clone CI:A3-1; Santa Cruz Biotechnology, Inc., Dallas, Texas, USA) or isotype control antibody (rat IgG2b, clone 141945; R&D Systems®, Inc., Minneapolis, MN, USA) for 48 h at 4 °C. Biotinylated goat anti-rat secondary antibody was used to detect the primary antibody and visualized using the ABC system with 3,3′-diaminobenzidine (DAB) as the chromogen (ImmunoCruz™ Rat ABC Staining System; Santa Cruz Biotechnology, Inc.). The sections were counterstained with Gill’s hematoxylin solution No. 2 (Santa Cruz Biotechnology, Inc.). Microscopic examination of macrophages in the peritoneal tissue was performed using an Olympus BX41 microscope. The ImmunoCruz™ Rat ABC Staining System was also used for immunohistochemical staining of blood vessels using rat anti-mouse PECAM-1/CD31 monoclonal antibody (clone RM0032-1D12; Santa Cruz Biotechnology, Inc.).

### Preparation for intravital microscopy

For all intravital microscopy (IVM) experiments, a new set of animals was used that was not treated with PD. The detailed methods on the surgical preparation for IVM were previously described [16]. Briefly, animals were anesthetized (ketamine and xylazine) and the right internal jugular vein was cannulated with a polyethylene catheter (ID 0.28 mm, OD 0.61 mm, Intramedic™, Becton, Dickinson and Company, Mississauga, ON, Canada) for maintenance of anesthesia, administration of fluids or fluorescent antibodies. Skin was bluntly dissected away and a midline incision along the linea alba was made in the abdominal wall extending inferiorly from the xiphoid process towards the left inguinal region. The animals were placed in the right lateral position and the peritoneum on the left side of the abdominal wall was laid out on a microscope stage and covered with plastic wrap to prevent evaporative loss.

### Spinning disk confocal intravital microscopy

Wild type mice were injected IP with *S. aureus* lipoteichoic acid (LTA) (125 μg; Sigma-Aldrich®) or a 100 μL suspension of GFP-expressing *S. aureus* cells containing approximately 1.8 × 10^8^ CFU (*n* = 4 mice). After 4 h, the mice were prepared for IVM of the parietal peritoneum microcirculation and injected intravenously (IV) with Alexa Fluor® 568-labeled rat anti-mouse syndecan-1 ectodomain antibody (40 μg, clone 300506; R&D Systems®, Inc.). The peritoneal microcirculation was visualized with a spinning disc confocal system (Leica DMI 6000 B; Leica Microsystems, Mannheim, Germany) based on a Yokogawa spinning disc confocal unit, spectral laser merge module LMM5, and Hamamatsu back-thinned electron multiplying charge-coupled device C9100-12 camera (Hamamatsu, Hamamatsu City, Japan). During the IVM observations, the animals were placed in a chamber mounted on the confocal microscope and the temperature in the chamber was set to 37 °C. The peritoneal microcirculation was observed with a 40× objective lens and images were acquired using the Volocity® 4 acquisition software (Improvision, Waltham, MA, USA).

### Quantification of fluorescence intensity

Using ImageJ (National Institutes of Health, W. Rasband, Bethesda, Maryland, USA), the fluorescence intensity of the Alexa Fluor® 568-conjugated anti-syndecan-1 antibodies was quantified from the captured *in vivo* images of peritoneal venules. The fluorescence intensity of the labeled antibodies was measured along the length of the basolateral side of the venular endothelium and the value for the corresponding intravascular fluorescence intensity was subtracted. This relative difference in intensity was calculated for 4 venules per mouse and the values were recorded as the difference in mean fluorescence intensity (arbitrary fluorescence units).

### Transillumination intravital microscopy

Wild type and *Sdc1*^-/-^ mice were injected IP with approximately 1.8 × 10^8^ CFU of *S. aureus* (100 μL), while clinical grade saline (0.9 % [w/v] NaCl) was injected IP into control animals (*n* = 4 mice). All IP injections were performed on the right side. The microcirculation underlying the parietal peritoneum on the left side of the abdominal wall was observed using an inverted intravital microscope (Zeiss Inverted Axiovert 100; Carl Zeiss, Jena, Germany). The tissue was transilluminated with a light source via fiberoptic (The ACE® Series; Schott-Fostec, LLC, Auburn, NY, USA) equipped with a 150 Watt tungsten halogen lamp. Images were captured with an attached camera (Newvicon; DAGE-MTI, Michigan City, IN, USA), projected onto a monitor (Panasonic, CT-2086YD; Panasonic Canada Inc., Mississauga, ON, Canada) and recorded with a DVD recorder (Panasonic, DMR-EH55) for offline analysis. The animals were warmed with an infrared heat lamp positioned over the microscope during the intravital observations. To minimize effects of tissue exposure during IVM [22], observations were made within 10 min after completion of surgical preparation. Euthanasia was performed by cervical dislocation under deep anesthesia.

### Offline analysis

Leukocyte-endothelial cell interactions were quantified in 4–6 venules per mouse. Rolling leukocytes, considered as cells tethering to a venule with torsional motion, were counted per minute. Intravascular leukocytes that remained stationary for at least 30 s were identified as adherent cells and perivenular leukocytes were counted as extravascular cells on a field of view measuring 180 μm × 135 μm.

### Statistical analysis

Data are expressed as mean ± standard error of the mean (SEM). Statistical significance was set at *p* < 0.05 and calculated using Student’s *t*-test or ANOVA with Bonferroni correction using the computer software package KaleidaGraph 3.6 (Synergy Software, Reading, PA, USA).

## Results

### Animal characteristics

Following catheter implantation surgery, there were no significant changes in behavior or appearance of the animals and skin incisions healed well, with no signs of infection. One mouse in the *Sdc1*^-/-^ group was euthanized at the end of the catheter acclimatization period because of fighting injuries from the cage mate. There were no differences in the weights of the animals between the wild type group and *Sdc1*^-/-^ group at the start (26.93 ± 0.30 g versus 25.88 ± 0.55 g, respectively) and end (30.50 ± 0.42 g versus 29.13 ± 0.44 g, respectively) of the experiment. Upon midline laparotomy, 1/14 wild type mice had omental wrapping of the catheter at the entry site on the abdominal wall, and 8/14 wild type mice and 5/8 *Sdc1*^-/-^ mice had gonadal adipose tissue wrapping at catheter entry site with no occlusion of the catheter tip.

### *Sdc1*^-/-^ mice have higher *S. aureus* loads in the abdominal wall after peritoneal dialysis

**Fig. 1:**
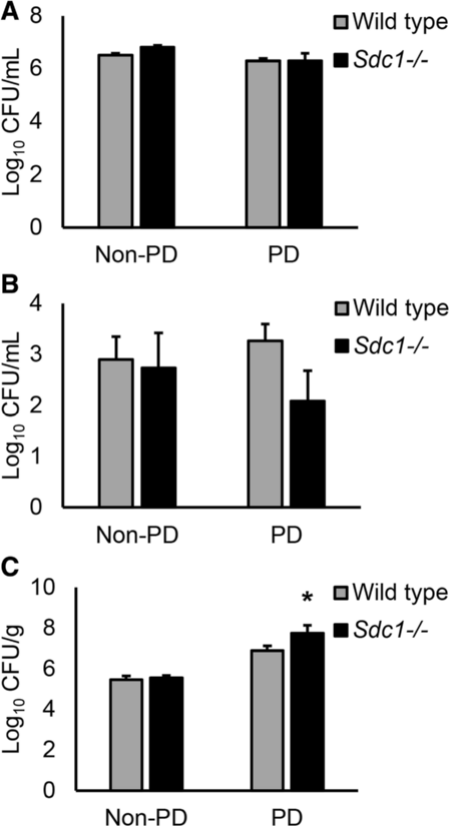
*Sdc1* ^-/-^ mice have higher *S. aureus* loads in the abdominal wall after peritoneal dialysis (PD). Viable bacteria counts from (**a**) lavage samples, (**b**) blood and (**c**) abdominal wall homogenates from wild type and *Sdc1* ^-/-^ mice collected 4 h after the animals were injected with 1.8 × 10^8^ colony-forming units (CFU) of *S. aureus* through the peritoneal catheter (PD group) or with an intraperitoneal needle injection (non-PD group). Data recorded as mean ± SEM, analyzed with ANOVA with Bonferroni correction and expressed as Log_10_ CFU per mL of peritoneal fluid or blood, or g of tissue, *n* = 14 mice in the wild type infected non-PD group and wild type PD group, *n* = 8 mice in all other groups, *p < 0.01 compared with all other groups. *Sdc1* ^-/-^ mice that were dialyzed for 4 weeks had a significantly higher number of viable bacterial cells in the abdominal wall samples after 4 h of infection

To determine whether subacute exposure to PD and lack of syndecan-1 affect the progression of *S. aureus* peritoneal infection, wild type and *Sdc1*^-/-^ mice were infected through the peritoneal catheter, while control animals were infected through a needle injection IP. Four hours post-infection, peritoneal lavage fluid, blood and a sample of the left abdominal wall were collected for CFU enumeration. There were no significant differences in the CFU counts from the peritoneal lavage between dialyzed animals and non-dialysis controls, or wild type and *Sdc1*^-/-^ mice (Fig. 1a). There were also no differences in CFU counts from blood samples between any of the groups (Fig. 1b). An outlier was excluded from the *Sdc1*^-/-^ PD group because the value was far beyond the range of values calculated by subtraction and addition of 1.5 times the interquartile range to the first and third quartile. Contamination during cardiac puncture was strongly suspected. Nevertheless, ANOVA tests with Bonferroni correction did not show any significant difference between the groups with and without the inclusion of the outlier. The bacterial counts from the blood suggested sepsis in some animals, which was accompanied by diarrhea and slower movement. The CFU counts from the abdominal wall homogenate samples were significantly increased in the *Sdc1*^-/-^ animals, but only in the group that was treated with PD (Fig. 1c). These results indicate that the parietal peritoneum and the underlying tissues of *Sdc1*^-/-^ mice are more susceptible to *S. aureus* invasion in the context of PD-induced injury.

All of the infected animals had a significantly increased number of peritoneal leukocytes compared with baseline levels (Table 1). Although the peritoneal leukocyte counts were reduced in the *Sdc1*^-/-^ animals with *S. aureus* infection compared with wild type, the difference was not statistically significant. Systemic white blood cell counts did not significantly differ between the groups; however, the number of systemic neutrophils was significantly decreased in the dialyzed wild type animals compared with non-PD controls. Also, the number of systemic monocytes was significantly elevated in wild type non-PD animals with *S. aureus* infection compared with *Sdc1*^-/-^ mice and baseline controls.

**Table 1:** Peritoneal lavage leukocyte counts and differential blood leukocyte counts

Treatment/strain	Total peritoneal cells × 10^6^	Total blood cells × 10^9^ L^-1^	Blood neutrophils	Blood lymphocytes	Blood monocytes
Baseline wild type	3.52 ± 0.43	4.08 ± 0.67	0.99 ± 0.27	3.06 ± 0.42	0.04 ± 0.01**
Baseline *Sdc1* ^-/-^	2.78 ± 0.28	4.56 ± 0.68	1.14 ± 0.26	3.40 ± 0.51	0.02 ± 0.01
Non-PD + *S. aureus* wild type	25.78 ± 3.43***	4.98 ± 0.65	1.80 ± 0.20	3.14 ± 0.53	0.34 ± 0.07
Non-PD + *S. aureus Sdc1* ^-/-^	15.94 ± 1.57***	3.62 ± 0.49	1.58 ± 0.31	2.01 ± 0.25	0.03 ± 0.02**
PD + *S. aureus* wild type	31.65 ± 1.90***	3.12 ± 0.33	0.79 ± 0.12*	2.14 ± 0.27	0.19 ± 0.04
PD + *S. aureus Sdc1* ^-/-^	22.42 ± 1.89***	3.29 ± 0.52	1.05 ± 0.16	2.11 ± 0.41	0.13 ± 0.03

At the end of the peritoneal dialysis (PD) period, mice were infected intraperitoneally with 1.8 × 10^8^ colony-forming units of *S. aureus* and 4 h after, peritoneal lavage was performed and blood was collected via cardiac puncture for enumeration of leukocytes. Data recorded as mean ± SEM and analyzed with ANOVA with Bonferroni correction, 6 counts averaged per mouse, *n* = 8–14 mice, *p < 0.01 and **p < 0.001 compared with non-PD wild type infected mice, ***p < 0.0001 compared with baseline values. *S. aureus*-infected mice had significantly increased numbers of peritoneal leukocytes compared with baseline but systemic leukocyte counts did not differ significantly

### Host material deposition in the peritoneal catheters of *Sdc1*^-/-^ and wild type mice

**Fig. 2:**
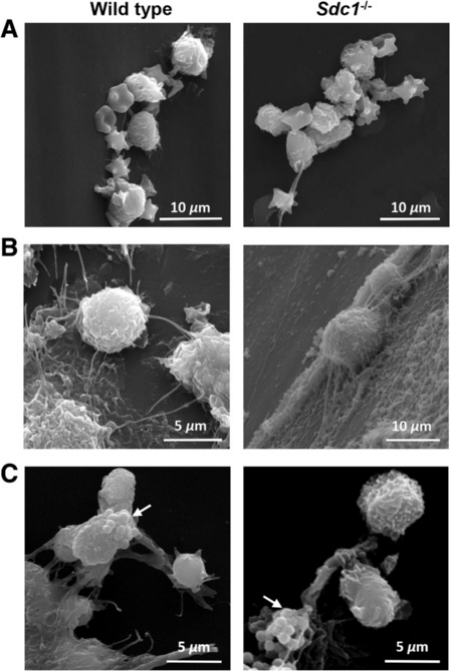
Host reaction to the peritoneal dialysis catheters. After 4 weeks of peritoneal dialysis, the intra-abdominal portion of the peritoneal catheter was collected from wild type and *Sdc1* ^-/-^ mice that were injected with *S. aureus* through the catheter. The catheters were fixed and prepared for scanning electron microscopy after 4 h of *S. aureus* infection. **a** Leukocytes and clotting components were recruited to the lumen of the catheter. Leukocytes were seen interacting with (**b**) other leukocytes and (**c**) *S. aureus* (arrows). Host responses to the biomaterial were similar in *Sdc1* ^-/-^ and wild type mice

Foreign body reactions are characterized by macrophage adherence to implants and fusion to form foreign body giant cells [23]. *In vitro* work showed that *Sdc1*^-/-^ macrophages have impaired motility [24]. Thus, foreign body reactions to the catheter implant were examined using scanning electron microscopy after 4 h of *S. aureus* infection. Leukocytes, platelets and crenated red blood cells were recruited to the catheter lumen (Fig. 2a). Leukocytes reacted to the foreign body by attachment to the surface within a fibrin network (Fig. 2b). These included macrophages and neutrophils that appeared to communicate through cell surface extensions. The inflammatory cells interacted with *S. aureus* microcolonies (Fig. 2c). The results suggest that syndecan-1 deficiency does not alter the recruitment of inflammatory cells to a foreign body.

### Syndecan-1 deficiency does not affect histopathologic changes of the peritoneum in subacute peritoneal dialysis

**Fig. 3:**
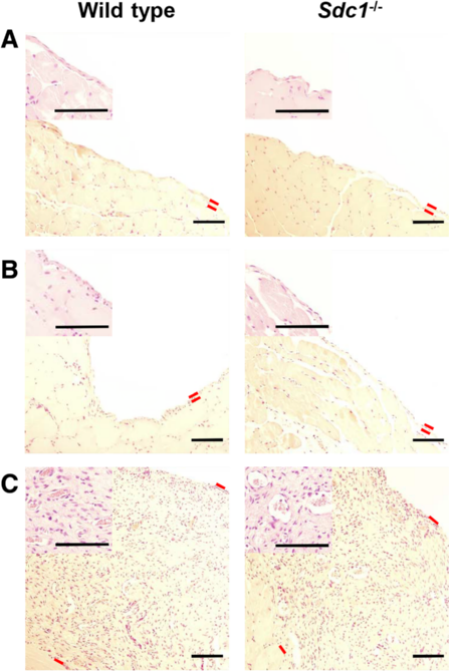
Severe peritoneal pathology in wild type and *Sdc1* ^-/-^ mice with subacute peritoneal dialysis. At the study endpoint, samples of the abdominal wall were collected from (**a**) wild type and *Sdc1* ^-/-^ mice that served as baseline controls, (**b**) wild type and *Sdc1* ^-/-^ mice that were injected with *S. aureus* intraperitoneally and (**c**) mice that were treated with peritoneal dialysis for 4 weeks and infected with *S. aureus* for 4 h. The samples were fixed, sectioned and stained with hematoxylin and eosin for histopathologic analysis. The peritoneal layer of dialyzed mice was thickened and is seen spanning the entire field of view. Red lines indicate boundaries of the peritoneal layer. Insets show increased vascularization of the submesothelial zone in dialyzed samples. Representative images, *n* = 14 mice in the wild type infected non-PD group and wild type PD group, *n* = 8 mice in all other groups, scale bar = 100 μm

**Fig. 4:**
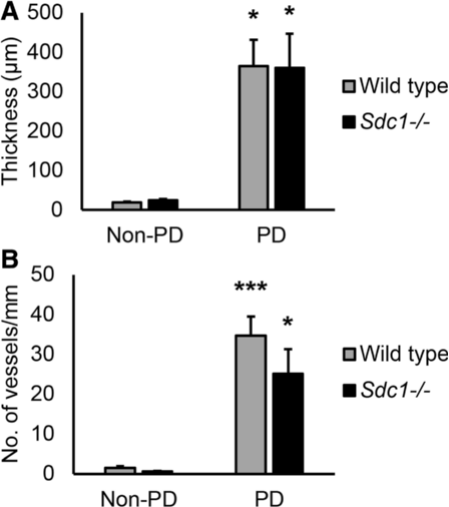
Wild type and *Sdc1* ^-/-^ mice exhibit similar levels of fibrosis and neovascularization during subacute peritoneal dialysis (PD). The (**a**) thickness and (**b**) vascular density of the peritoneal layer were quantified from the hematoxylin and eosin-stained sections. Data recorded as mean ± SEM and analyzed with ANOVA with Bonferroni correction, *n* = 14 mice in the wild type groups and *n* = 8 mice in the *Sdc1* ^-/-^ groups, *p < 0.01 and ***p < 0.0001 compared with controls that were not treated with peritoneal dialysis (non-PD). There were no differences in the levels of fibrosis and vascularization in the peritoneal layer of wild type and *Sdc1* ^-/-^ mice

**Fig. 5:**
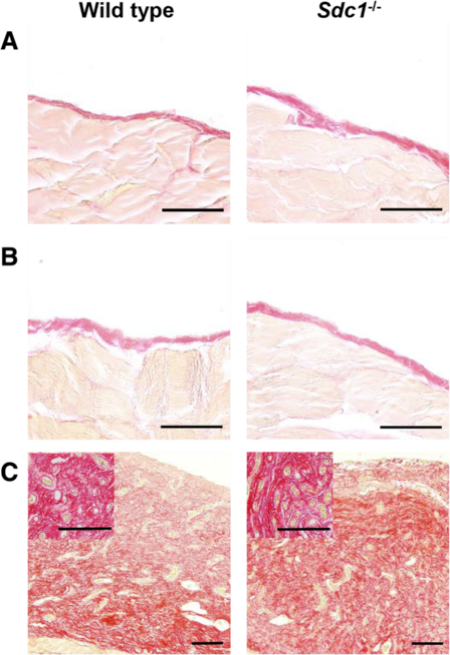
Extensive collagen deposition in wild type and *Sdc1* ^-/-^ mice with subacute peritoneal dialysis. At the study endpoint, samples of the abdominal wall were taken from (**a**) baseline control mice, (**b**) undialyzed animals that were injected with *S. aureus* and (**c**) mice that were treated with peritoneal dialysis for 4 weeks and infected with *S. aureus* 4 h before the endpoint. The sections were stained with picro-sirius red to visualize collagen fibers. The thickened peritoneal layer of dialyzed mice was rich in collagen fibers that were disorganized in both, the wild type and *Sdc1* ^-/-^ mice. Representative images, *n* = 14 mice in the wild type infected non-PD group and wild type PD group, *n* = 8 mice in all other groups, (**a **& **b**) scale bar = 50 μm and (**c**) scale bar = 100 μm

**Fig. 6:**
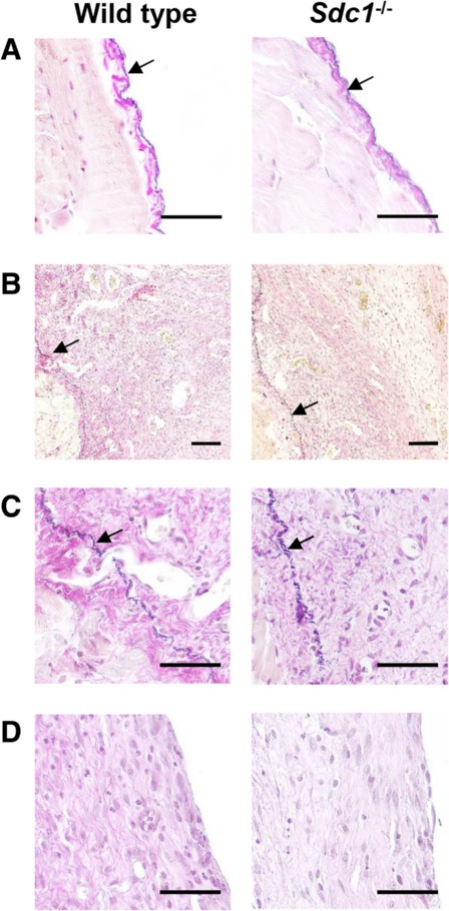
Similar changes in elastic tissue in wild type and *Sdc1* ^-/-^ mice with subacute peritoneal dialysis. Sections of the abdominal wall from (**a**) *S. aureus*-infected undialyzed control mice and (**b**) *S. aureus*-infected animals that were treated with peritoneal dialysis for 4 weeks were stained with resorcin-fuchsin and counterstained with van Gieson’s solution and Weigert’s iron hematoxylin to identify elastic fibers (indicated by arrows). **a** The network of elastic fibers was near the mesothelial surface in the undialyzed animals but was far below the mesothelial surface in the (**c**) dialyzed wild type and *Sdc1* ^-/-^ animals. **d** Elastic fibers were not observed near the mesothelial surface in the dialyzed animals. Representative images, *n* = 14 mice in the wild type groups and *n* = 8 mice in the *Sdc1* ^-/-^ groups, (**a, c **& **d**) scale bar = 50 μm and (**b**) scale bar = 100 μm

**Fig. 7:**
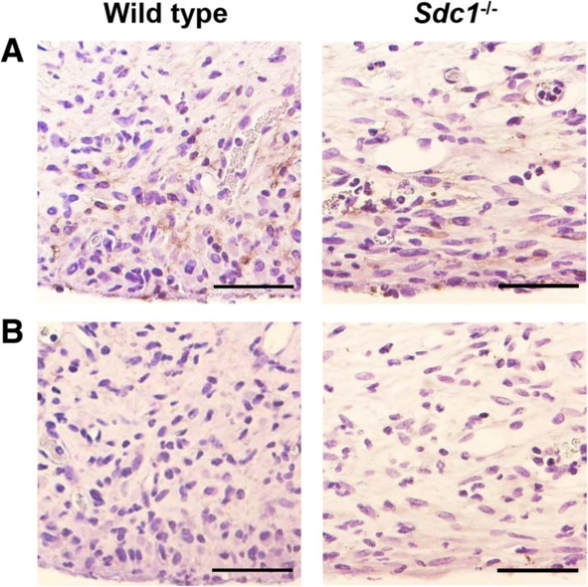
Macrophages concentrate near the peritoneal surface in wild type and *Sdc1* ^-/-^ mice with subacute peritoneal dialysis. Sections of the abdominal wall from *S. aureus*-infected animals that were treated with peritoneal dialysis for 4 weeks were prepared for immunohistochemistry and incubated with (**a**) anti-F4/80 antibody to identify macrophages or (**b**) the isotype control antibody and counterstained with Gill’s hematoxylin No. 2. In some areas of the sections, F4/80^+^ cells were found in the fibrotic peritoneum concentrated near the surface (brown color). Representative images, *n* = 4 mice, scale bar = 50 μm

Baseline control animals had an intact mesothelial surface (Fig. 3a) and non-PD mice that received an IP injection of *S. aureus* did not exhibit mesothelial disruption (Fig. 3b). Mice that were treated with PD, however, had a thickened peritoneum, with increased vascularization, interstitial hypercellularity, and leukocyte infiltration (Fig. 3c). The average thickness of the peritoneal layer was significantly increased in the PD groups (Fig. 4a), which suggests fibrosis. However, there was no significant difference between *Sdc1*^-/-^ mice and wild type animals. The number of peritoneum-associated vessels was significantly increased in dialyzed animals compared with non-PD controls but did not differ between the *Sdc1*^-/-^ mice and wild type animals (Fig. 4b). These blood vessel counts were verified with immunohistochemically-stained paraffin sections with anti-PECAM-1/CD31 and were found to be similar between the two methods (data not shown). Increased collagen deposition is characteristic of fibrosis. Extensive and disorganized collagen deposition was observed in the thickened peritoneal layer of dialyzed animals (Fig. 5) in both wild type and *Sdc1*^-/-^ mice. While the submesothelial layer of elastic fibers was near the mesothelial surface in undialyzed animals (Fig. 6a), it was several cell layers below the surface in the dialyzed wild type and *Sdc1*^-/-^ mice (Fig. 6b-d). Macrophages are a source of profibrotic molecules [25]. In the parietal peritoneum, depletion of macrophages was associated with decreased fibrosis in a mouse model of PD [26]. Thus, the presence of macrophages in the fibrotic peritoneal tissue was detected with IHC using F4/80, a cell surface marker for mature macrophages in mice. With the immunohistochemical analysis, F4/80^+^ cells were not detected in the infected non-PD control animals, but were found to be concentrated near the peritoneal surface in the dialyzed wild type and *Sdc1*^-/-^ animals in some areas of the sections (Fig. 7a). The adjacent sections that were incubated with the isotype control antibodies had no significant background signal (Fig. 7b). Taken together, these findings suggest that syndecan-1 is not a major modulator of morphological changes due to fibrosis and angiogenesis in PD-induced histopathology.

### *S. aureus* infection decreases subendothelial syndecan-1 in the peritoneal venules

**Fig. 8:**
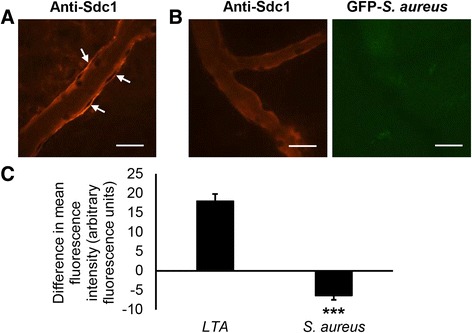
*S. aureus* infection decreases subendothelial syndecan-1 levels of the peritoneal venules. Wild type mice were injected intraperitoneally with (**a**) 125 μg of lipoteichoic acid (LTA) from *S. aureus* or (**b**) 1.8 × 10^8^ colony-forming units of green fluorescent protein (GFP)-expressing *S. aureus*. After 4 h, the mice were prepared for fluorescence intravital microscopy and Alexa Fluor® 568-labeled anti-syndecan-1 antibodies were injected intravenously. The microcirculation of the parietal peritoneum was examined. Syndecan-1 was detected in the subendothelial compartment of venules in (**a**) LTA-stimulated mice (arrows) but not with (**b**) *S. aureus* infection. Representative images, scale bar = 20 μm. **c** Quantification of fluorescence intensity from the antibodies labeling the subendothelial surface showed that syndecan-1 protein expression during *S. aureus* infection was significantly decreased. Data recorded as mean ± SEM and analyzed with Student’s *t*-test, 4 venules averaged per mouse, *n* = 4 mice, ***p < 0.0001 compared with LTA

To examine the effects of *S. aureus* infection on syndecan-1 levels in the peritoneal microcirculation, fluorescence IVM was performed on wild type mice that were injected IP with *S. aureus* LTA or GFP-expressing *S. aureus* and, after 4 h, the mice were injected IV with Alexa Fluor® 568-labeled anti-syndecan-1. During LTA-induced sterile inflammation, anti-syndecan-1 was detected on the subendothelial side of peritoneal venules (Fig. 8a). With *S. aureus* infection, however, anti-syndecan-1 antibody did not label peritoneal venules (Fig. 8b), as shown by quantification of fluorescence intensity from the anti-syndecan-1 antibodies on venules in LTA-injected and *S. aureus*-infected mice (Fig. 8c). The GFP-expressing *S. aureus* cells were not visible against the tissue background (Fig. 8b). These findings indicate that *S. aureus* infection decreased syndecan-1 protein levels in the subendothelial region of peritoneal venules.

### Syndecan-1 does not modulate leukocyte recruitment to the parietal peritoneum microcirculation during *S. aureus* infection

**Fig. 9:**
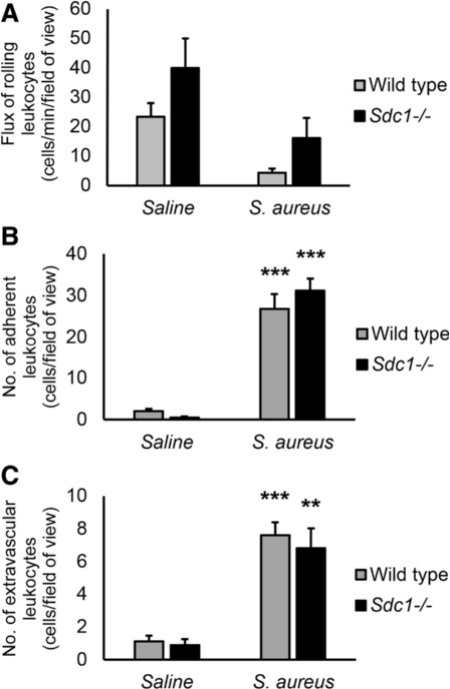
Syndecan-1 does not modulate leukocyte recruitment to the parietal peritoneum microcirculation in the early response to *S. aureus* infection. Wild type and *Sdc1* ^-/-^ mice were injected intraperitoneally with 1.8 × 10^8^ colony-forming units of *S. aureus*. After 4 h, the microcirculation of the parietal peritoneum was imaged with intravital microscopy. The numbers of (**a**) rolling and (**b**) adherent leukocytes in the peritoneal venules, as well as the number of (**c**) extravascular leukocytes, were quantified. *S. aureus* infection significantly increased the number of adherent and extravascular leukocytes compared with saline controls. However, there were no significant differences in leukocyte recruitment between the wild type and *Sdc1* ^-/-^ mice. Data recorded as mean ± SEM and analyzed with ANOVA with Bonferroni correction, 4–6 venules averaged per count, *n* = 4 mice, **p < 0.001 and ***p < 0.0001 compared with saline

To investigate whether syndecan-1 deficiency alters leukocyte recruitment during *S. aureus* infection, wild type and *Sdc1*^-/-^ mice were injected with a suspension of *S. aureus* IP and prepared for IVM after 4 h. Control mice were injected IP with sterile saline. Numbers of rolling leukocytes in mice infected with *S. aureus* were similar to saline controls (Fig. 9a). The number of adherent (Fig. 9b) and extravascular (Fig. 9c) leukocytes was significantly increased in *S. aureus* infection. However, there were no significant differences in the numbers of rolling, adherent and extravascular leukocytes between wild type and *Sdc1*^-/-^ mice. Thus, syndecan-1 does not modulate leukocyte trafficking in peritoneal venules during *S. aureus* infection.

## Discussion

*Sdc1*^-/-^ mice were reported to have abnormal phenotypes in injury and infection models [11, 17, 27–29]. In this study, we examined whether the abnormal phenotypes associated with syndecan-1 deficiency occur in PD. We observed that histopathologic alterations of the peritoneum did not differ between *Sdc1*^-/-^ and wild type mice. However, *Sdc1*^-/-^ mice were more susceptible to *S. aureus* infection, but only in the context of PD and despite the fact that syndecan-1 deficiency did not affect leukocyte trafficking in *S. aureus* peritonitis. Thus, although syndecan-1 did not affect histopathology in PD, it was important for decreasing *S. aureus* dissemination.

Our observation that *Sdc1*^-/-^ mice were more susceptible to *S. aureus* colonization of the abdominal wall after 4 weeks of PD contrast with findings in the corneal tissue, where *Sdc1*^-/-^ mice cleared *S. aureus* infection more effectively while *S. aureus* induced syndecan-1 shedding from the corneal epithelium in wild type animals [17]. Other studies also found protective effects of syndecan-1 deficiency during infections. Syndecan-1 deficiency protected mice in *P. aeruginosa* lung infection [30], and this pathogen was also found to promote syndecan-1 shedding. As well, *Sdc1*^-/-^ mice had decreased mortality, less systemic bacterial dissemination and decreased cytokine production compared with wild type animals in a burn-wound model with *P. aeruginosa* infection [31]. One difference, however, is that these studies used injury models that are acute in nature, whereas our study involved 6-week exposure to a peritoneal catheter and 4-week exposure to dialysis solution. Perhaps the ability of pathogens such as *S. aureus* to subvert syndecan-1 shedding to enhance pathogenesis is decreased in longer injury models. This idea raises the question whether *Sdc1*^-/-^ mice in this long-term injury model would have similarly increased susceptibility to other pathogens, such as *P. aeruginosa*, during an acute peritoneal infection. Given that *P. aeruginosa* infection in syndecan-1 deficiency results in attenuated pro-inflammatory cytokine responses [31], it is plausible that increased susceptibility to this pathogen would also be observed in the *Sdc1*^-/-^ mice with the subacute PD model during the first 4 h of infection where pro-inflammatory cytokines orchestrate the innate immune response to pathogen invasion.

The cytoplasmic domain of syndecan-1 binds cytoskeletal proteins and PDZ-domain proteins and, consequently, regulates remodelling of the actin cytoskeleton [32]. Syndecan-1 also mediates cell adhesion through its effects on integrins [33]. Not surprisingly, syndecan-1 deficiency was reported to impair epithelial barriers in injury and inflammation. After corneal wounding, *Sdc1*^-/-^ mice had decreased re-epithelialization, reduced α_9_-integrin localization and failure of apical repolarization of the tight junction protein zonula occludens (ZO)-1 [11]. Also, *Sdc1*^-/-^ mice had decreased intestinal epithelial barrier function resulting in increased intestinal protein leakage, a finding that was replicated in human epithelium *in vitro* [34]. Disruption of epithelial barriers is important in *S. aureus* pathogenesis. For example, in human airway epithelium, *S. aureus* caused disruption of actin and ZO-1 [35]. In this study, *S. aureus* activated proteolysis of junctional proteins to alter the epithelial barrier for transepithelial invasion. In human intestinal epithelium, *S. aureus* α-toxin disrupted the barrier integrity with a reduction of cellular junctional proteins, namely ZO-1, ZO-3 and E-cadherin [36]. Thus, another possibility is that the mesothelial layer of the *Sdc1*^-/-^ mice in our study was more easily breached by *S. aureus*, resulting in increased invasion of the abdominal wall with subacute PD-induced injury. As well, given that decreased epithelial integrity mediates pathogen invasion in wounds, it is plausible that syndecan-1 deficiency during PD-induced injury would also lead to increased susceptibility to other pathogens such as *P. aeruginosa*, a common cause of wound infections [37].

Although syndecan-1 was shown to be involved in angiogenesis, in that *Sdc1*^-/-^ mice exhibited increased corneal neovascularization in an angiogenesis assay [28] and syndecan-1 overexpression promoted tumor angiogenesis [12], we found that the increased vessel density in dialyzed animals was similar between *Sdc1*^-/-^ and wild type mice. Furthermore, we found no evidence that fibrotic responses and collagen organization were altered in dialyzed *Sdc1*^-/-^ mice compared with wild type, even though altered phenotypes were noted by others in fibrotic mouse models. For example, in a mouse model of myocardial infarction, *Sdc1*^-/-^ mice had elevated leukocyte recruitment and increased matrix metalloproteinase activity with increased collagen fragmentation and disorganization [29]. This phenotype also involved increased cardiac dilation and dysfunction. In an angiotensin II-induced cardiac fibrosis mouse model, however, *Sdc1*^-/-^ mice exhibited less cardiac fibrosis with decreased expression of collagen type I and III and reduced cardiac dysfunction [13]. Thus, the effects of syndecan-1 deficiency differ based on the fibrosis model. In transforming growth factor-β1-induced peritoneal fibrosis, a secondary basement membrane was formed near the mesothelial surface, while remnants of the original basement membrane were found 50 to 100 μm below the mesothelial surface in the rat peritoneum [38]. The peritoneal basement membrane is lined by a network of elastic fibers [39]. Our findings show that while the original layer of elastic fibers was several cell layers below the surface, a new network of elastic fibers was not formed near the mesothelial surface in PD, and this fibrotic response was not affected by syndecan-1 deficiency. As well, *Sdc1*^-/-^ and wild type mice both had heterogeneous distribution of macrophages in the fibrotic tissue that were mostly concentrated near the peritoneal surface. Peritoneal fibrosis in PD models was shown to be largely due to a foreign body reaction to the catheter implant that contained a sterile cell layer of red blood cells, leukocytes, mesothelial cells and fibroblasts [40]. Thus, the catheter acts as an inflammatory source in the peritoneum. Host material deposition and leukocyte recruitment to the catheter lumens were evident in our study, and the foreign body responses to the catheter biomaterial were similar between *Sdc1*^-/-^ mice and wild type animals.

Previously, we found that syndecan-1 levels do not change in the parietal peritoneum microcirculation in response to *S. aureus* LTA [16], as LTA-challenged mice had subendothelial syndecan-1 levels that were similar to the saline-treated animals even though LTA induced leukocyte recruitment in the peritoneal microcirculation. This suggests that “sterile” inflammation does not promote syndecan-1 shedding. However, syndecan-1 shedding was repeatedly shown to be induced by pathogens [30, 41], such as *S. aureus* [17], and the microbial components that activate syndecan-1 shedding from cellular membranes were deduced to be *S. aureus* α- and β-toxins [42]. These toxins induced syndecan-1 shedding by activating the host cell’s protein-tyrosine kinase-dependent shedding mechanisms [42]. In line with these results, we observed that 4 h after IP injection of a suspension of viable *S. aureus* cells, syndecan-1 levels in the subendothelial region of peritoneal venules were significantly diminished. Inflammatory cytokines regulate the expression of syndecan-1 at the protein and mRNA levels [43]. Therefore, in addition to the pathogen-induced shedding mechanisms, it is plausible that syndecan-1 expression in our study was affected by cytokines released in response to the *S. aureus* infection. Together, these findings indicate that *S. aureus* LTA-induced inflammation in the peritoneum does not model the inflammatory responses to viable *S. aureus* cells.

Although we observed dysentery and bacteremia, the *S. aureus* infection did not result in severely lowered systemic white blood cell counts that were seen in the cecal ligation and puncture model of sepsis [44]. Also, we did not see severely diminished leukocyte recruitment to peritoneal venules that was observed in the endotoxemia model of sepsis [16] and the mouse cremaster microcirculation during IP *S. aureus* infection [45]. However, in the cremaster muscle study, the dose of live *S. aureus* cells was higher than used in our study. We observed greater leukocyte recruitment in acute *S. aureus* infection in this study compared with the response to IP injection of *S. aureus* LTA observed in our previous work. Also, similar to our previous observations on peritoneal leukocyte recruitment in response to LTA, lipopolysaccharide or tumour necrosis factor-α (TNFα), there were no differences in leukocyte recruitment between wild type animals and *Sdc1*^-/-^ mice during infection [16]. Others reported that syndecan-1 deficiency in mice is associated with increased leukocyte recruitment in TNFα-stimulated retinal and mesenteric microcirculation [28], naïve cremasteric venules [46], and during experimental autoimmune encephalomyelitis [47], delayed-type hypersensitivity response [48], and anti-glomerular basement membrane nephritis [49]. These different findings emphasize the uniqueness of the molecular mechanisms that regulate leukocyte recruitment in the parietal peritoneum. However, an important consideration is that *in vitro* work indicated that *Sdc1*^-/-^ monocytes may be the dominant cell type in the increased leukocyte infiltrate [50], and higher numbers of monocytes are usually recruited *in vivo* after 3 to 4 days following peritonitis [51], which may explain our observations that *Sdc1*^-/-^ mice do not have elevated leukocyte recruitment in the early response to infection. Thus, it should be emphasized that our conclusions regarding the effects of syndecan-1 on infection and inflammation are only relevant to the early acute inflammatory responses after 4 h of infection.

## Conclusions

Our study demonstrated that syndecan-1 deficiency does not impact histopathologic deterioration of the peritoneal layer in a mouse model of PD. Thus, despite the clinical relevance of syndecan-1 in the prognosis of diseases such as multiple myeloma [52], and sepsis [53], syndecan-1 does not appear to be important in PD-induced pathology. We did, however, find that syndecan-1 is important for limiting acute peritoneal *S. aureus* infection, which may have implications for incidence of peritonitis and infections in PD patients. Alongside current literature, the seemingly contrasting observations emphasize that syndecan-1 functions are cell- and tissue-specific and call for a careful comparison between experimental models. Furthermore, future research should dissect the peritoneal responses to *S. aureus* at longer infection time points during syndecan-1 deficiency and compare these responses with other major pathogens that cause peritonitis in PD in order to fully understand the role of syndecan-1 in injury, infection and inflammation.
